# Serum cell-free DNA and progression of diabetic kidney disease: a prospective study

**DOI:** 10.1136/bmjdrc-2019-001078

**Published:** 2020-03-09

**Authors:** Xuan Li, RenZhi Hu, Ting Luo, Chuan Peng, Lilin Gong, Jinbo Hu, Shumin Yang, Qifu Li

**Affiliations:** 1 Department of Endocrinology, the First Affiliated Hospital of Chongqing Medical University, Chongqing, Chongqing, China; 2 The Chongqing Key Laboratory of Translational Medicine in Major Metabolic Diseases, the First Affiliated Hospital of Chongqing Medical University, Chongqing, China

**Keywords:** cfDNA, diabetes, chronic kidney disease, prospective study

## Abstract

**Aims:**

Cell-free DNA (cfDNA) is associated with diabetes and cardiovascular diseases. Our study was to evaluate whether serum cfDNA could predict the progression of diabetic kidney disease (DKD).

**Methods:**

In this prospective study, a total of 160 patients with DKD were enrolled, and the kidney function was followed up by measurement of estimated glomerular filtration rate (eGFR) and urinary albumin–creatinine ratio (UACR) for three consecutive years. At baseline, concentrations of serum cfDNA were measured. DKD progression was defined as two-continuous decrease in eGFR and changes of UACR from less than 300 mg/g at baseline to higher than 300 mg/g at last follow-up. Regression models were used to analyze associations of serum cfDNA with the DKD progression.

**Results:**

In total, 131 patients finished all the follow-up visits. At the end of the study, 64 patients showed decreased eGFR and 29 patients had changes of UACR from less than 300 mg/g at baseline to higher than 300 mg/g at follow-up. At baseline, the progression group had higher serum cfDNA levels than the non-progression group (960.49 (816.53, 1073.65) ng/mL vs 824.51 (701.34, 987.06) ng/mL, p=0.014). Serum cfDNA levels were significantly negatively associated with the 1.5-year eGFR change (r=−0.219 p=0.009) and 3-year eGFR change (r=−0.181, p=0.043). Multivariate logistic analyses showed that after adjustment of age, gender, body mass index, fast plasma glucose, smoking, triglycerides, total cholesterol, duration of diabetes, systolic blood pressure, diabetic retinopathy, eGFR, high sensitivity C-reactive protein, angiotensin receptor blocker/ACE inhibitor usage, with the increase of one SD of serum cfDNA levels, the risk of DKD progression increased by 2.4 times (OR, 2.46; 95% CI 1.84 to 4.89).

**Conclusion:**

Serum cfDNA is closely associated with DKD, and it might be a predictor of DKD progression in patients with type 2 diabetes.

**Significance of this study**

**What is already known about this subject?**

- Serum cell-free DNA (cfDNA) levels have been reported to be elevated in patients with diabetes, especially in patients with diabetic retinopathy, implying a potential relationship between cfDNA and diabetic microvascular complication.

**What are the new findings?**

- Serum cfDNA is closely associated with diabetic kidney disease (DKD), and it might be a predictor of DKD progression in patients with type 2 diabetes.

**How might these results change the focus of research or clinical practice?**

- Future study might be focused on the causal relationship between cfDNA and DKD and whether cfDNA is biomarker for early diagnosis of DKD.

## Introduction

With the increasing incidence of type 2 diabetes (T2D), diabetic kidney disease (DKD) is becoming a worldwide public health problem. Developing a non-invasive surrogate marker that can reflect the extent of progression of DKD is urgently needed.^1^ Identification of pathophysiologically important markers also helps to discriminate those patients at high risk for progression to end stage renal disease and then treat them timely and effectively. Other than the traditional risk factors such as age, hypertension, urine protein, and estimated glomerular filtration rate (eGFR), whether other non-traditional factors could serve as potential predictors of poor kidney outcome is worthy of investigation.

As a genetic material, DNA is mainly found in the nucleus. Cell-free DNA (cfDNA) refers to fragmented DNA that is free of extracellular cells and is present in body fluids such as blood, urine, synovial fluid, and cerebrospinal fluid. cfDNA is derived from cell necrosis, apoptosis, and autonomic release following cellular synthesis of nucleic acids.^2^ Serum cfDNA levels were found to be elevated in patients with diabetes, and among patients with diabetes serum cfDNA levels were higher in patients with retinopathy than those without retinopathy.^3^ In addition, the elevation of cfDNA in patients with diabetes with DKD was more pronounced as compared with patients without DKD^4^

The aims of this study were to evaluate the association of serum cfDNA with the changes in eGFR or albuminuria and to explore whether serum cfDNA could predict the progression of DKD.

## Materials and methods

### Study design

This was a prospective observational study. Patients with DKD were recruited from 2014 February to 2017 February in the endocrinology department of the First Affiliated Hospital of Chongqing Medical University based on the inclusion criteria: (1) 18–70 years of age; (2) T2D diagnosis based on blood glucose test or self-reported diabetes history which was validated by previous medical records and treatment with antidiabetic agents; (3) spot urinary albumin to creatinine ratio (UACR) of >30 mg/g for twice in 3 months, with other influence factors such as infection excluded. Patients diagnosed with other chronic kidney diseases were excluded. Patients were followed up for 3 years.

### Sample size calculation

Power Analysis and Sample Size software V.11 (PASS 11) was used to calculate the sample size. In the procedure of logistic regression, the main outcome (DKD progression after 3-year follow-up) was used as a binary response variable (Y), and the baseline serum cfDNA concentration was used as a continuous variable (X). The event rate of DKD Progression was assumed to be 15% among patients with DKD, so P0 (baseline probability that Y=1) was set as 0.15. Based pilot data, the OR (Odds1/Odds0) was assumed as 2.0. After running the program of logistic regression, a sample size of 130 observations was suggested to be large enough to detect an OR of 2.0 with 81% power at the 0.05 significance level with a two-sided test. Considering a 20% missing rate during the follow-up, a sample size of 160 subjects was needed. At baseline, we finally recruited 160 subjects with DKD and conducted this prospective study.

### Laboratory methods

Plasma glucose levels were measured with a hexokinase glucose-6-phosphate dehydrogenase method by biochemical analyzer (BS-380; Mindray Medical International, Shenzhen, China). Serum total cholesterol (TC), triglycerides (TG), high-density lipoprotein cholesterol (HDL-c), and low-density lipoprotein cholesterol (LDL-c) were measured enzymatically on an automatic analyzer (Model 7080; Hitachi, Tokyo, Japan) with reagents purchased from Leadman Biochemistry Co. (Beijing, China). Serum creatinine and cystatin C were measured with the use of an automatic biochemical analyzer (Modular DDP, Roche).

A commercially available kit (Q33120, USA, Thermo), which was described in a previous study,^5^ was used to determine the concentrations of serum cfDNA. All experimental procedures were carried out according to the manufacturer’s instructions. Renal function was measured as eGFR calculated by the Modification of Diet in Renal Disease. We tested two indices of change in eGFR as done in previous studies.^6^ Progression of DKD was defined as two-continuous decreases in eGFR and changes of UACR from less than 300 mg/g at baseline to higher than 300 mg/g at follow-up.

### Definitions of outcomes

The “1.5-year eGFR change” was defined as eGFR result of 1.5-year follow-up visit minus baseline eGFR. The “3-year eGFR change” was defined as eGFR result of 3-year follow-up visit minus baseline eGFR. The “1.5-year UACR change” was defined as UACR result of 1.5-year follow-up visit minus baseline UACR. The “3-year UACR change” was defined as UACR result of 3-year follow-up visit minus baseline UACR. Progression of DKD was defined as two-continuous decreases in eGFR (1.5-year eGFR was lower than baseline eGFR and 3-year eGFR was lower than 1.5-year eGFR) or changes of UACR from less than 300 mg/g at baseline to higher than 300 mg/g at 3-year follow-up.

### Statistics analysis

Variables distributed normally were presented as mean±SD, while variables with skewed distribution were presented as medians (IQR) and analyzed after logarithmic transformation. Categorical variables were reported as frequencies or proportions and were analyzed by χ^2^ test. Pearson correlation analyses were used to test the correlations between individual variables, and multiple linear regression analyses with the change in eGFR as dependent variable and serum cfDNA as independent variables, respectively. Models were built to adjust for confounding factors. Multivariate logistic analyses were conducted to detect the relationship between serum cfDNA and risk of eGFR decline; several models were built to adjust for confounding factors. Age, duration of diabetes, systolic blood pressure (SBP), HbA1c combined with or without cfDNA were included in multiple logistic regression models to predict DKD progression, and the area under the receiver operating curves (AUCs) were, respectively, calculated based on the predictive varieties.

## Results

**Figure 1:**
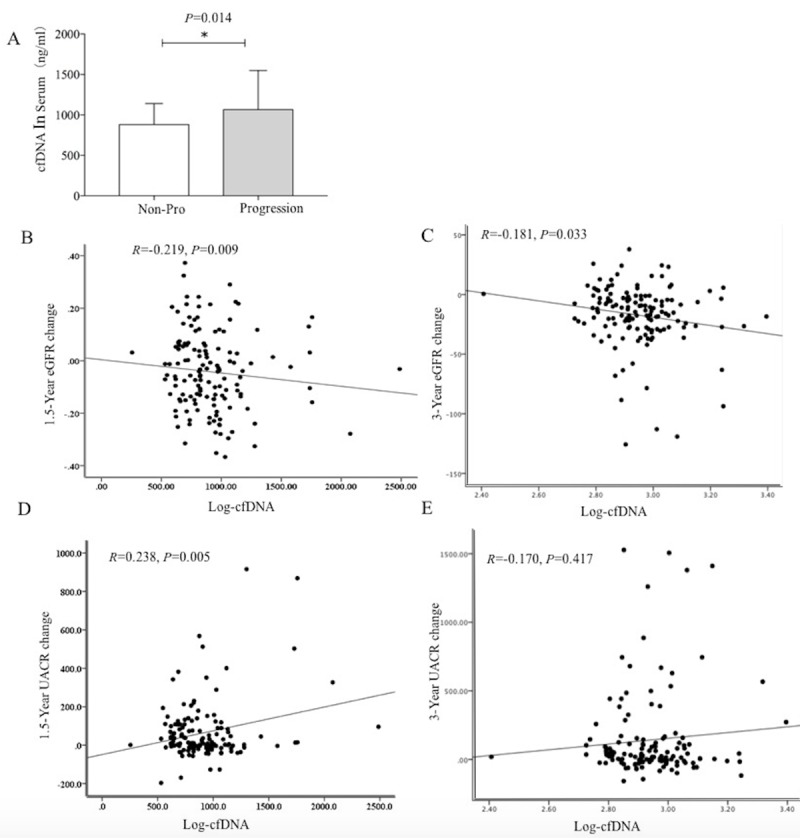
(A) The serum cfDNA level in non-progression group and progression group at baseline. The p value was calculated with the use T test. (B) The simple correlation analysis of the serum log-cfDNA level with 1.5-year eGFR change. (C) The simple correlation analysis of the serum log-cfDNA level with 3-year eGFR change. (D) The simple correlation analysis of the serum log-cfDNA level with 1.5-year UACR change. (E) The simple correlation analysis of the serum log-cfDNA level with 3-year UACR change. cfDNA, cell-free DNA; eGFR, estimated glomerular filtration rate; UACR, urinary albumin–creatinine ratio

In total, 160 subjects with DKD were enrolled in this study and 131 patients finished all the follow-up visits. During the follow-up, 64 patients showed two-continuous decrease in eGFR and 29 patients had the change of UACR from less than 300 mg/g at baseline to higher than 300 mg/g at the end of the study. The baseline characteristics of the subjects are shown in table 1. There were 73 men and 58 women. The mean age of subjects was 62.30±6.37 years. The mean duration of diabetes was 11.23±6.03 years. The mean eGFR was 90.81±24.47 mL/min/1.73 m^2^ (table 1). The serum cfDNA concentration in the progression group was significantly higher than that in the non-progression group (960.49 (816.53 to 987.06) vs 824.51 ng/mL (701.34 to 987.06), p=0.014) (figure 1A). Those who did not finish the study did not differ from those who finished the study (online supplementary table 1).

10.1136/bmjdrc-2019-001078.supp1 Supplementary data

**Table 1:** Baseline characteristics of metabolic and laboratory parameters

Variable	Total	Non-Pro	Progression	P value
Men/women (person)	73/58	61/52	12/6	/
Age (year)	62.30±6.37	62.47±6.63	61.22±6.50	0.456
Duration of diabetes	11.23±6.03	11.10±5.80	9.66±5.58	0.327
BMI (kg/m^2^)	26.09±3.07	25.98±3.13	26.66±2.86	0.387
WC (cm)	97.32±6.93	89.98±8.15	90.78±78.07	0.700
Smokers (%)	48.85	32.53	44.40	0.000
HT history (%)	60.89	69.71	79.02	0.316
ARB/ACEI usage(%)	93.75	99.11	96.39	0.618
FPG (mmol/L)	7.91±2.43	7.78±2.41	7.96±2.61	0.772
TC (mmol/L)	4.11±0.99	4.15±1.03	3.83±0.67	0.022
TG (mmol/L)	2.01±2.05	2.05±2.22	2.07±1.39	0.964
SBP (mm Hg)	136.34±16.34	135.82±16.34	134.36±12.13	0.716
DBP (mm Hg)	74.27±10.26	74.48±10.46	77.39±7.67	0.262
Creatinine (umol/L)	72.51±22.01	71.93±21.57	79.50±19.50	0.164
BUN (mmol/L)	6.00±1.68	5.91±1.65	6.85±1.49	0.024
UA (umol/L)	354.46±100.94	344.50±85.40	402.11±103.90	0.115
UACR (mg/g)	75.55±76.67	61.48±67.33	136.00±84.29	0.000
eGFR (mL/min per 1.73 m^2^)	90.81±24.47	91.22±25.09	82.72±19.61	0.173

DKD progression was defined as two-continuous decrease in eGFR and changes of UACR from less than 300 mg/g at baseline to higher than 300 mg/g at follow-up.

ACEI, ACE inhibitor; ARB, angiotensin receptor blocker; BMI, body mass index; BUN, blood urea nitrogen; DBP, diastolic blood pressure; eGFR, estimated glomerular filtration rate; FPG, fast plasma glucose; HT history, hypertension history; SBP, systolic blood pressure; TC, triglyceride; TG, total cholesterol; UA, uric acid; WC, waist circumference.

In the simple correlation analysis, serum cfDNA was significantly negative associated with the 1.5-year eGFR change (r=−0.21, p=0.009) (figure 1B) and with the 3-year eGFR change (r=−0.18, p=0.033) (figure 1C). In the simple correlation analysis, serum cfDNA was significantly associated with the 1.5-year UACR changes (r=0.23, p=0.005) (figure 1D). However, there is no significant association between cfDNA and the 3-year UACR changes (r=−0.17, p=0.417) (figure 1E).

In the multiple linear regression analyses, with the adjustment of age, gender, duration of T2D, SBP, body mass index (BMI), fast plasma glucose (FPG), TG, HDL-c, LDL-c, baseline serum cfDNA concentration showed independent association with the changes in 1.5-year eGFR change (β=−0.17, p=0.049) and 3-year eGFR change (r=−0.17, p=0.049) (table 2).

**Table 2:** Multiple linear regression analysis for the correlation between serum cfDNA and the decline of kidney function

	Crude		Adjusted	
	Standard β	P value	Standard β	P value
1.5-year eGFR change	−0.18	0.024	−0.17	0.049
3-year eGFR change	−0.18	0.023	−0.17	0
1.5-year UACR change	0.23	0.005	0.18	0.055
3-year UACR change	0.15	0.083	0.09	0.289

Adjusted for age, gender, duration of Type 2 diabetes, systolic blood pressure, body mass index, fast plasma glucose, triglycerides, high-density lipoprotein-cholesterol; low-density lipoprotein-cholesterol.

cfDNA, cell-free DNA; eGFR, estimated glomerular filtration rate; UACR, urinary albumin–creatinine ratio.

In the multivariate logistic analyses, adjusting for, age, gender, BMI, FPG, smoking, TG, TC, duration of diabetes, SBP, diabetic retinopathy, eGFR, high sensitivity C-reactive protein, angiotensin receptor blocker/ACE inhibitor usage, with the increase in one SD of serum cfDNA levels, the risk of DKD progression increased by 2.4 times (OR, 2.46; 95% CI 1.84 to 4.89) (table 3).

**Table 3:** Multivariates logistic regression analysis for DKD progression

Model	DKD progression	
	Or (95% CI)	P value
Crude	1.89 (1.11 to 3.21)	0.017
Multiple	2.46 (1.84 to 4.89)	0.021

Adjusted for age, gender, body mass index, fast plasma glucose smoking, triglycerides, total cholesterol, duration of diabetes, systolic blood pressure, DR, estimated glomerular filtration rate, high sensitivity C-reactive protein, ACE inhibitor/angiotensin receptor blocker usage.

DKD, diabetic kidney disease; DR, diabetic retinopathy.

Using the traditional model including age, duration of diabetes, SBP, HbA1c, to predict DKD progression, the AUC was 0.61 (95% CI 0.53 to 0.69). While when cfDNA was added to the traditional model, the AUC increased to 0.65 (95% CI 0.57 to 0.73).

## Discussion

CfDNA has been detected in plasma, serum, urine, and other body fluids from healthy subjects as well as in patients.^7^ Circulating cfDNA has now been shown to be useful in the diagnosis or monitoring of diseases such as trauma stroke,^8^ myocardial infarction,^9^ and tumor.^10^ In this prospective study, we provided important evidence of the association between serum cfDNA and progression of DKD. Higher serum cfDNA concentrations were proved to be associated with the annual and percentage decline in eGFR. Noteworthy, the relationship was independent of known risk factors of DKD, including age, duration of diabetes, blood pressure, and glucose.

It was reported that high glucose stimulation can increase the production of extracellular DNA in neutrophils.^11^ Human study found that the level of serum cfDNA in patients with diabetes is significantly higher than that in normal subjects. Furthermore, among patients with diabetes the serum cfDNA in DKD group is higher than non-DKD group. Our findings are consistent with previous studies above. In addition, serum cfDNA levels in patients with diabetic retinopathy were higher than that in patients without retinopathy. These studies suggest that cfDNA may be associated with diabetes and its microvascular complications.^4^

In our study, we found that there is no significant relationship between 3-year UACR progression, and it may because UACR is an early marker of renal endothelial cell dysfunction. Although our study observed an association between serum cfDNA and DKD, the mechanism behind this phenomenon was unclear. A recently published study analyzed the role of cfDNA in acute kidney injury induced by ischemia.^12^ The renal injury was associated with higher intra-renal DNA debris. More importantly, administration of exogenous deoxyribonuclease ameliorated cfDNA-induced damage and resulted in the improvement of renal perfusion and functions.^13^ This indicates that cfDNA is not only a consequence of renal tissue damage but also amplify the damaging processes in acute kidney injury. In vitro, cfDNA from the plasma of hemodialysis patients induces interleukin six production in human monocytes, suggesting a pro-inflammatory effect of cfDNA.^14^ Besides, extracellular DNA combining with granular proteins (eg, histones) formed neutrophil extracellular traps which have been demonstrated to be detrimental in the pathogenesis of some renal disease, such as anti-neutrophil cytoplasmic antibodies (ANCA)-associated glomerulonephritis, lupus nephritis, and septic acute kidney injury.^15^ But whether cfDNA or a complex of cfDNA and protein is involved the development of DKD needs further study.

## Limitations

The current study was limited by a relatively small population and short follow-up time and we did not measure cfDNA in the follow-up visits. Prospective studies with larger sample size and DNase intervention are needed to further evaluate the contribution of cfDNA in the development of DKD.

## Conclusion

In conclusion, we found that elevated serum cfDNA concentrations were independently associated with an increased risk of DKD progression. Serum cfDNA might be a predictor of DKD in patients with T2D. Further studies are needed to replicate these findings and to explore potential mechanisms underlying the observed association.
