# Supplementary material for: Serum cell-free DNA and progression of diabetic kidney disease: a prospective study
Source: BMJ Open Diabetes Res Care. 2020 Mar 9;8(1):e001078. doi: 10.1136/bmjdrc-2019-001078 (PMC7064129; doi:10.1136/bmjdrc-2019-001078)
Supplement: Supplementary data [file bmjdrc-2019-001078supp001.pdf]

Supplementary Table 1. Baseline characteristics of the subjects completed and uncompleted the study

Variable	Completed (n=131)	Uncompleted (n=29)	P value
Men/women (person)	73/58	16/13	0.411
Age (year)	62.30±6.37	62.31±6.38	0.697
Duration of diabetes	11.23±6.03	11.03±6.03	0.834
BMI (kg/m^2)	26.09±3.07	26.19±3.07	0.653
WC (cm)	97.32±6.93	97.41±6.92	0.521
Smokers (%)	48.85	45.75	0.578
HT history (%)	60.89	60.85	0.895
ARB/ACEI usage(%)	93.75	93.79	0.642
FPG(mmol/l)	7.91±2.43	7.881±2.43	0.484
TC(mmol/l)	4.11±0.99	4.11±1.00	0.693
TG(mmol/l)	2.01±2.05	2.01±2.06	0.821
SBP(mmHg)	136.34±16.34	136.30±16.69	0.533
DBP(mmHg)	74.27±10.26	74.46±10.30	0.631
Creatinine(μmol/L)	72.51±22.01	71.33±22.31	0.486
Bun(mmol/L)	6.00±1.68	5.98±1.68	0.454
UA(μmol/L)	354.46±100.94	355.84±101.41	0.383
UACR(mg/g)	75.55±76.67	75.85±77.04	0.499
eGFR (ml/min per 1.73 m^2)	90.81±24.47	90.81±24.56	0.526
cfDNA(ng/mL)	871.69(722.38,1027.51)	871.71(722.60,1027.54)	0.166

BMI: body mass index. WC: waist circumference. HT history: hypertension history. FPG: fast plasma glucose. TC: triglyceride. TG: total cholesterol. SBP: Systolic blood pressure. DBP: Diastolic blood pressure. BUN: blood urea nitrogen. UA: Uric acid eGFR: estimated glomerular filtration rate. cfDNA: cell free DNA.
